# Sensitivity Analysis and Uncertainty Quantification of Nanoparticle Deposition from Tongue Morphological Variations

**DOI:** 10.3390/life14030406

**Published:** 2024-03-19

**Authors:** Tiancheng Yang, Xiuhua Si, Jinxiang Xi

**Affiliations:** 1 Department of Mechanical Engineering, Northwestern University, Evanston, IL 60208, USA; tianchengyang2019@u.northwestern.edu; 2 Shanghai Rebo Granulator Co., Ltd., 489 Gangde Road, Shanghai 201100, China; 3 Department of Aerospace, Industrial and Mechanical Engineering, California Baptist University, Riverside, CA 92504, USA; asi@calbaptist.edu; 4 Department of Biomedical Engineering, University of Massachusetts, Lowell, MA 01854, USA

**Keywords:** tongue posture, orally inhaled drug products, pulmonary drug delivery, surrogate model, sensitivity analysis, uncertainty quantification

## Abstract

The human tongue has highly variable morphology. Its role in regulating respiratory flows and deposition of inhaled aerosols remains unclear. The objective of this study was to quantify the uncertainty of nanoparticle deposition from the variability in tongue shapes and positions and to rank the importance of these morphological factors. Oropharyngeal models with different tongue postures were reconstructed by modifying an existent anatomically accurate upper airway geometry. An LRN k-ω model was applied to solve the multiregime flows, and the Lagrangian tracking approach with near-wall treatment was used to simulate the behavior and fate of inhaled aerosols. Once the database of deposition rates was completed, a surrogate model was trained using Gaussian process regression with polynomial kernels and was validated by comparing its predictions to new CFD simulations. Input sensitivity analysis and output updateability quantification were then performed using the surrogate model. Results show that particle size is the most significant parameter in determining nanoparticle deposition in the upper airway. Among the morphological factors, the shape variations in the central tongue had a higher impact on the total deposition than those in the back tongue and glottal aperture. When considering subregional deposition, mixed sensitivity levels were observed among morphological factors, with the back tongue being the major factor for throat deposition and the central tongue for oral deposition. Interaction effects between flow rate and morphological factors were much higher than the effects from individual parameters and were most significant in the throat (pharyngolaryngeal region). Given input normal variances, the nanoparticle deposition exhibits logarithmical normal distributions, with much lower uncertainty in 100-nm than 2-nm aerosols.

## 1. Introduction

High dosimetry variability in orally inhaled products (OIPs) presents a significant obstacle to accurate dose-outcome correlation [1]. Despite rapid advances in computational drug delivery in recent years, variability and uncertainty in predictive dosimetry remain largely unquantified. A reliable dosimetry prediction requires accurate representations of the respiratory system, breathing conditions, and pharmaceutical properties [2]. In practice, various sources of uncertainty exist for these inputs, potentially leading to a strong bias in predicted dosages [3,4,5,6]. It is particularly important to consider these uncertainties when selecting and assessing regulatory options. Variability in pulmonary drug delivery can result from a variety of factors, including anatomical variations among subjects, breathing conditions, device use, and drug properties [4,5,6]. It may not be possible to fully control these factors, which may result in substantial uncertainty about the efficacy and outcomes of drug delivery. Furthermore, most prior predictive inhalation dosimetry studies have been deterministic, failing to account for the significant variability intrinsic to inhalation therapy [7,8,9]. There is a growing need for better analysis methods that can provide more useful information than conventional deterministic ones and, at the same time, are computationally efficient in providing real-time feedback.

Previous studies have focused on nanoparticle properties, delivery devices, and delivery parameters, such as particle size, plume angle, exiting velocity, head position, and breathing maneuver. Selmani et al. [10] systemically reviewed the synthesis, characterization, and stability of nanoparticles. Lu et al. [11] performed a probabilistic analysis to quantify input sensitivity and output uncertainty with variability in three input parameters (particle diameter, particle density, and inhalation speed), each having a normal distribution, with the variance σ being 20% of the mean value µ. The major setback of probabilistic analysis is the requirement of large numbers of numerical tests (i.e., Monte Carlo simulations), thus being prohibitively time-consuming and resource-demanding. Studies on the effects of geometry variations on inhalation dosimetry have been significantly scarce, primarily due to the higher technical challenges in generating airway models representative of the anatomy and physiology under different conditions. Recent progress in imaging and segmentation algorithms has made it possible to create individualized models from CT and MRI scans. However, 3D rendering of the respiratory tract is still hindered by the low resolutions of the scans, which often lead to unrealistic geometries with artifacts [12,13,14]. As a result, previous studies often included only a limited number of patient-specific models [15,16]. To gain a deeper understanding of intersubject variability, it is necessary to understand better the influences of airway morphological details on the behavior and fate of orally inhaled particles [17,18].

The tongue position or posture during inhalation drug delivery is often unnoticed by both the patients and caregivers. The tongue acts as an impaction surface for the high inertia of orally inhaled drug particles. However, numerical or experimental studies dedicated to the effects of tongue position are scarce, presumably due to the technical challenges involved in generating representative airway models with different tongue postures. Zhou et al. [19] considered the effect of the inhaler’s position on the distribution of orally administered medications within the mouth-throat geometry, reconstructed from CT images of four pediatric patients. Although not explicitly stated, the tongue positions varied in these four models, leading to notable differences in front oral cavity volume, oral airway curvature, and minimal oral passage area. These factors further caused differences in flow recirculation intensity in the anterior oral cavity, inertia deposition on the tongue surface, and deposition on the mouth roof and throat. Wedel et al. [20] reported that the anatomical details of the upper airway could significantly alter the particle deposition distribution, specifically in the throat, which was consistent with a previous study by Xi and Longest [21]. On the other hand, the impact of the flow rate on the deposition pattern was insignificant, appearing similar under varying flow rates [20]. Xi and Yang [22] numerically considered the effects of tongue morphological variations on orally inhaled micron particles (1–10 µm) in the oropharyngeal airway. The tongue’s position exerted a notable impact on respiratory flow and particle deposition patterns in the mouth-throat geometry, leading to a 6–25% variability in filtration efficiency contingent upon the breathing condition and particle size.

The delivery of medication through orally inhaled pulmonary systems, such as inhalers or nebulizers, is a complex process influenced by multiple factors. These include breathing patterns, the particle size of the medication, and the geometry of the respiratory tract. The interaction among these factors introduces complexity or nonlinearity in inhalation dosimetry, making it challenging to predict drug delivery efficiency accurately. The combination of factors can lead to outcomes that are not predictable by considering each factor in isolation. For example, a particular combination of particle size and breathing condition may lead to higher dosage in a specific lung region due to synergistic effects that would not be evident when analyzing these factors separately. Certain conditions may need to be met before a significant change in deposition occurs. For nanoparticles, the deposition profile vs. particle size exhibits a U-shape, first decreasing from 0.5 nm to 100 nm (ultrafine) due to decreasing diffusivity, then increasing from 200 nm (fine) with increasing inertia [23]. The effect of inhalation rate also differs in this process, where a higher flow rate reduces ultrafine particle deposition by shortening their residence time but increases the deposition of large nanoparticles by increasing their inertia [24]. Furthermore, the flow’s influencing power varies, being inversely proportional to residence time and squared for inertia. Interactions with geometrical factors can be significantly more complex and thus difficult to quantify accurately. Understanding the complex interactions among these factors is crucial for optimizing pulmonary drug delivery systems.

The objective of this study is to leverage surrogate modeling and computational fluid dynamics (CFD) to examine a complex issue in orally inhaled drug delivery, i.e., the effect of tongue morphologies on inhalation dosimetry. There are five tasks in this study:

1. Developing oropharyngeal models with varying tongue positions.
2. Conducting high-fidelity CFD simulations to build a database.
3. Preprocessing the database to ensure balanced data representation.
4. Training the surrogate model and validating it against new CFD predictions.
5. Conducting input sensitivity analysis and dosimetry uncertainty quantification.

## 2. Materials and Methods

### 2.1. Mouth-Throat Models with Varying Tongue Postures

**Figure 1:**
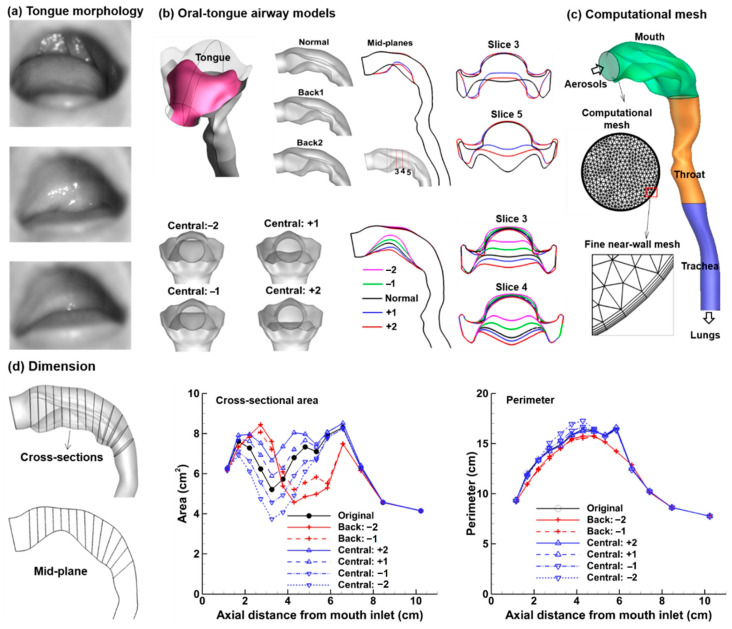
Morphology and dimensions of the oropharyngeal models with varying tongue shapes and positions: (**a**) high-speed images of various tongue positions, (**b**) oral-tongue (pink) models with morphological modifications at the back tongue (i.e., Back 1 and Back 2) and central tongue (i.e., Central: −2, −1, +1, +2) visualized both in 3D and mid-sagittal cross-sections, (**c**) the oropharyngeal geometry with three compartments (mouth, pharynx, and trachea) and a high-quality computational mesh, and (**d**) the dimensions of different oral-tongue models in terms of cross-sectional area and perimeter.

To generate different tongue positions (Figure 1a), HyperMorph (Troy, MI, USA) was used to modify an existing mouth-lung geometry. Briefly, the region of interest (ROI) was split into several sections, with handles specified at the boundary nodes of each section, like a scaffold surrounding a building. Moving these handles changed the overall shape of the scaffold, which, in turn, modified the morphology of the airway model within. By moving the local handles in a controlled, quantitative manner, the surface geometry of the tongue could be tuned to an intended shape and size. As shown in Figure 1b, the tongue morphology was changed in the middle and at the back. Due to geometrical constraints, the back tongue was only raised by two levels (i.e., Back 1 and Back 2 in the upper panel in Figure 1b). The central tongue was modified in both directions, i.e., being raised by two levels (Central: −1 and Central: −2, reducing the central oral airway) and tucked by two levels (Central: +1 and Central: +2, expanding the central oral airway), as displayed in the lower panel of Figure 1b. The computational mesh was correspondingly deformed (Figure 1c). The dimensions of modified models are compared in Figure 1d, which shows clear differences in the cross-sectional areas at the middle and back of the tongue. The differences in the volume and surface area among different models are listed in Table 1.

### 2.2. Study Design

**Figure 2:**
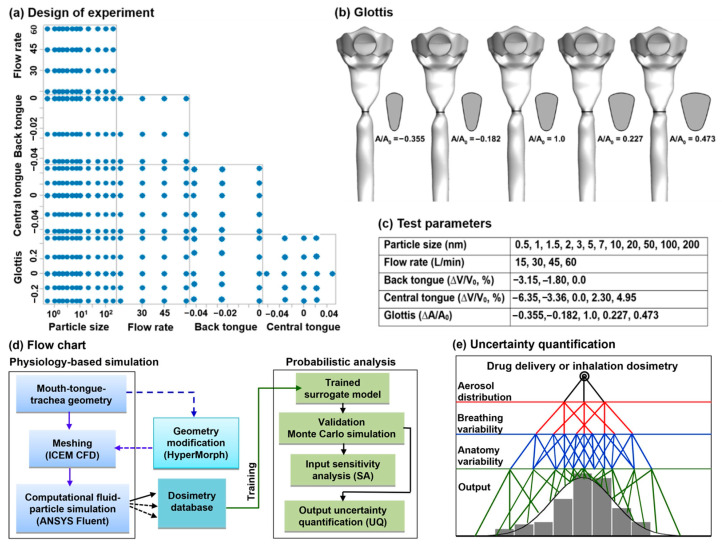
Study design and flow chart: (**a**) test cases with varying input parameters: breathing (flow rate), aerosols (particle size), and anatomy (back tongue, central tongue, and glottal aperture), (**b**) glottis models with five different apertures, (**c**) the design space of test parameters, (**d**) flow chart outlining the surrogate modeling process, and (**e**) diagram of uncertainty quantification.

To understand the effect of individual factors, a scatter matrix of test cases was planned, as displayed in Figure 2a, depicting the variation and pairing of various influential factors such as breathing (flow rate), aerosols (particle size), and anatomy (back tongue, central tongue, and glottal aperture). Each column or row corresponds to one of the variables in the study, with the intersections where they meet showing scatter plots of the paired relationships between them. Looking at the particle size in the first column, one can observe how the particle size interacts with other variables such as the glottis, tongue position, or flow rate. A denser distribution of points for ultrafine particles reflects the greater variability of deposition in this particle size range. Similarly, for the flow rate in the second column (or first row), the spread of data points across different values of anatomical variables (back tongue, central tongue, and glottis) could indicate how particle size interacts with anatomical factors to affect deposition. Specifically, the glottis variants are demonstrated in Figure 2b, while the design space of all test parameters is listed in Figure 2c. The particle size ranges from 0.5 nm to 200 nm (Figure 2c). The lower end represents chemical vapors [25], while the upper end represents nanoparticles that can escape macrophage, thus minimizing mediation clearance or degradation [26].

Figure 2d shows a flow chart for physiology-based CFD simulations and surrogate modeling-based probabilistic analysis. This first part involves reconstructing mouth-tongue-trachea geometrical models, meshing, conducting case-specific deposition simulations, HyperMorph-enabled geometry modification, and database development. In the second part, a surrogate dosimetry predictive model will be developed using the kernel-based Gaussian process regression (GRP) method. The trained model will be validated and then applied to perform Monte Carlo simulations, which rely on repeated random sampling to understand how input uncertainty affects the outcomes. Sensitivity analysis is conducted to determine how sensitive the results are to changes in each input parameter, thus identifying which variables have the most significant impact on the outcome and are, therefore, critical to the accuracy of the model. The final step is uncertainty quantification, which determines the uncertainty in the simulation outputs due to the input variability/uncertainty. This is crucial for understanding the confidence level of the simulation results and for making informed decisions based on those results. In this study, the propagation of uncertainty is illustrated in Figure 2e, where variability/uncertainty in aerosol distribution, breathing, and the respiratory airway interact to determine the uncertainty in the predicted dosimetry.

### 2.3. CFD Simulations for Database Development

Constant inhalation rates ranging from 15 to 60 L/min were examined, assuming ambient pressure at the inlet and vacuum pressures at the trachea. The low Reynolds number (LRN) k-ω model was employed to resolve the inspiratory flows, which were assumed to be incompressible and isothermal [27]. The LRN k-ω model has been demonstrated to deliver precise results for laminar flow conditions as the turbulent viscosity reduces to zero [28]. A well-tested, discrete-phase Lagrangian tracking approach was applied to track particle motion and deposition. User-defined functions (UDFs) were developed that considered the finite inertial effects of particles [29]. The nanoparticles evaluated in this study had a diffusivity ranging from 2.17 × 10^−1^ cm^2^/s (0.5 nm) to 2.29 × 10^−6^ cm^2^/s (200 nm). Dilute aerosols were assumed, with no impact on the airflows (i.e., one-way coupling). The influence of Brownian motion on particle paths was accounted for at each time step as a distinct force per unit mass term following the formula proposed by Li & Ahmadi [30]. This Lagrangian tracking approach, with a near-wall interpolation UDF, has provided a good match with in vitro and in vivo measurements in the respiratory tract for both nanoparticles and micrometer-sized particles [31].

ANSYS Fluent 21 (Canonsburg, PA, USA) was utilized to simulate the airflow and particle deposition. The computational meshes were generated using ANSYS ICEM CFD (Ansys, Inc.). A mesh sensitivity analysis was performed by changing the computational mesh from very coarse to ultrafine until the change in deposition fractions between two consecutive runs reduced below 1% [32]. The final computational mesh consisted of 1.8 million cells. There were five layers of prismatic elements near the wall, and the height of the first layer cell was 0.05 mm. Particle sensitivity analysis was conducted for 2-nm aerosols by incrementally increasing the number of seed particles by 10,000 from 30,000 until there was no significant variation between consecutive tests. An amount of 100,000 particles was found to generate statistically invariant deposition predictions, which was used in all subsequent CFD simulations.

### 2.4. Surrogate Model Training, Validation, and Uncertainty Quantification

SmartUQ 6.3 (Madison, WI, USA) was utilized for surrogate modeling, sensitivity analysis (SA), and uncertainty quantification (UQ). The goal of developing a surrogate model was to detect the complex input-output relationships specific to inhalation dosimetry in the upper airway. An accurate surrogate model was the foundation for subsequent space exploration, design optimization, sensitivity analysis, and uncertainty propagation/quantification. The Gaussian process regression (GPR) method was employed to train the kernel-based surrogate model (also called an emulator or meta-model) [33],

(1) $$y_{0}=\sum_{i=1}^{n}w_{i}k(x_{i},\;x_{0})$$

where *k*(*x_i_*, *x*_0_) is the kernel function measuring the similarity between any pair of inputs, and *w_i_* is the kernel function weight to be learned from the training dataset comprising 290 test cases. The Gaussian process (GP) is a nonparametric method used in supervised learning to address regression and probabilistic classification issues [34]. The core idea behind GP is to place a prior probability distribution over functions, enabling the modeling of functions with uncertainty in a Bayesian framework (updating beliefs based on new evidence or information). Kernels are predefined variance functions of data transformations, which enable the surrogate model to capture inherent nonlinear relationships and allow data separations that are not linearly separable in the original input space. Polynomial kernels that computed data similarity as a polynomial function of the dot product in the feature space were used in this study, allowing for quantification of interactions between input factors [35,36]. To validate the trained surrogate model, surrogate predictions were compared with ten new CFD cases. The operating conditions of the test cases were generated using the design of experiment method, allocating sampling points for a maximal design space coverage [37,38].

The relative significance of tongue posture (sensitive analysis) was subsequently assessed against other factors, such as inhalation velocity and particle diameter. Some 100,000 cases were sampled, and the importance of each input variable was quantified. Input sensitivity was quantified in terms of both the main effect and total effect, with the former being the effect of a single input on the output by ignoring all other inputs, while the total effect encompassing both the main effect and the effects from interactions with other inputs. The uncertainty in deposition was also quantified based on 100,000 sampling points using the surrogate model. It estimated the propagation of input uncertainty to the simulation outputs. It is noted that the Gaussian process prediction is a distribution, not a number, which is different from conventional correlations (1D) or response surfaces (2D) [39,40,41].

## 3. Results

### 3.1. Airflow and Particle Deposition Simulations: Developing Datase

#### 3.1.1. Airflow

**Figure 3:**
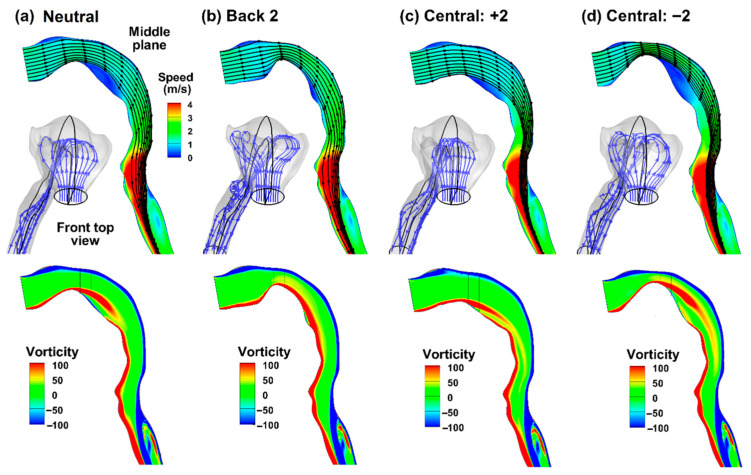
Inspiratory airflow streamlines (upper panels) and vorticity (lower panels) in the oropharyngeal airway: (**a**) neutral position, (**b**) Back 2 (back-raised), (**c**) Central: +2 (central-tucked), and (**d**) Central: −2 (central-raised). The streamlines are seeded at identical positions at the mouth inlets but exhibit very different trajectories due to the changing tongue positions.

Figure 3a–d shows the inspiratory airflows in the oropharyngeal airway at 30 L/min with a normal glottis configuration (i.e., A/A_0_ = 1.0, Figure 2b), with the tongue having a neutral, back-raised, central-tucked, and central-raised position, respectively. The tongue acted as a rounded blockage to the inspiratory flow by rising from the mouth floor, thus elevating the streamlines towards the mouth roof, affecting the inertia of the airflow approaching the back of the mouth (oropharynx). Figure 3a–d shows that the tongue position notably altered the flow patterns. In a neutral position (Figure 3a), the streamlines initially followed an uprising curve of the front tongue, accelerating and spreading laterally towards the teeth-cheek lumens. The airflows then moved down toward the back of the mouth, leaving a recirculation zone behind the tongue (blue region) due to the abrupt tucking of the caudal tongue. At the oropharynx, the airflow streamlines underwent a 90° direction change from horizontal to vertical while converging to form an approximate circular flow, creating a zone of high inertia loss for inhaled particles. The flow subsequently moved through the pharynx and throat, continuing to accelerate and reaching maximal speed at the glottis, which generated a laryngeal jet in the downstream trachea (red, Figure 3a).

With the tongue raised towards the back (Back 2, Figure 3b), the oral cavity is constricted. The tighter space caused the streamlines to bend more sharply around the tongue, resulting in higher speeds as the air passes the constriction. One unexpected observation was that the raised back tongue effectively eliminated flow recirculation, as shown in Figure 3b. The secondary lateral flows were also more pronounced, with more airflows passing through the teeth-cheek lumen. In Figure 3c, the tongue was tucked down at the center, leading to a wider path and slower airflow through the central region, as indicated by the more blue-green in this region. The flow recirculation noted in a neutral tongue position (Figure 3a) was also reduced. The ten streamlines seeded from the horizontal middle line of the mouth (blue, bottom view) showed negligible lateral motions. On the other hand, the ten streamlines seeded from the vertical middle line (black, lateral view) are more concentrated in the throat (red arrow, Figure 3c), indicating a sophisticated interaction between the airflow and structure, as well as the interplay between airflow streamlines seeded from different regions of the mouth inlet. In Figure 3d, with the raised central tongue, the streamlines are more compressed and accelerated in the central region, as suggested by the less uniform spaces between streamlines. A more extensive recirculation zone formed downstream of the central tongue due to a sharp slope. The streamlines seeded from the vertical middle line are concentrated in the throat (black, lateral view), similar to, or even more pronounced than, the central-tucked case (Figure 3d vs. Figure 3c). Taking all other tongue positions into consideration, this similarity may indicate a common feature associated with the central tongue. Different from the central-raised condition (Figure 3c), streamlines in the middle horizontal (blue, Figure 3d) are more laterally spread due to compression by the raised tongue.

The lower panel of Figure 3 shows the flow vorticity in the middle sagittal plane of the oropharyngeal airway. Two shear layers formed near the wall, denoted in blue along the upper boundary and red along the lower boundary. Separations of the shear layers were apparent when the tongue assumed a neutral or central-raised position (Figure 3a,d) and appeared insignificant when the tongue assumed a back-raised or central-tucked position (Figure 3b,c). Particularly in Figure 3b, the convex shape of the back-raised tongue nearly eliminated the flow separation, allowing a smooth, continuous boundary layer in the oral cavity. Flow separations were also observed in the upper trachea (blue), which appeared similar among the four models considered.

#### 3.1.2. Nanoparticle Dynamics

**Figure 4:**
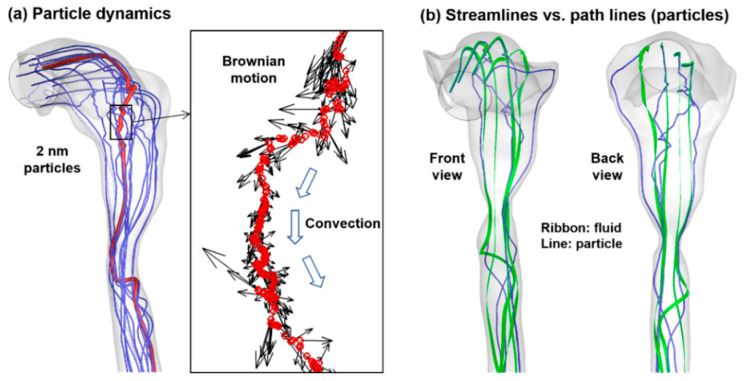
Nanoparticle dynamics: (**a**) path lines of 2-nm particles under the influence of convection and diffusion (Brownian motion). (**b**) Airflow and aerosol particles can have different trajectories, even when released from identical locations. Blue: particles; green ribbon: airflow.

Figure 4a displays the trajectories of ten 2-nm diameter particles randomly seeded at the mouth inlet, with wiggling motions in the oropharynx and swirling, twisted motions in the throat. The inset in Figure 4a shows the zoomed view of the dynamics of one specific particle in the back of the oropharynx, where the red circles depict the time series locations at consequential time steps. The random motions are apparent, characterized by zig-zag deviations from the main flow stream’s curvature (i.e., convection) and chaotic variations in vector directions and magnitudes (Brownian motion). The random motions appear more evident in the back of the tongue than in other regions due to the relatively slow speed and/or flow recirculation, making the Brownian motion of 2-nm particles more dominant than the convection effects.

Perceivable differences are noted between the airflow streamlines (green ribbons) and particle path lines (blue lines), as illustrated in Figure 4b. This is because of the quicker response time of the continuous airflow vs. the integrative nature of discrete particles, where the instantaneous flow direction is determined by the local pressure. In contrast, the particle path is a cumulative result of its history and the local flow. Understanding where and how the flow and particles separate can provide valuable insight into the behavior and fate of inhaled particles. Flow-particle separations were observed in the back mouth and upper trachea; the former had abrupt geometry changes, and the latter was preceded by a laryngeal jet, amplifying any existent differences.

**Figure 5:**
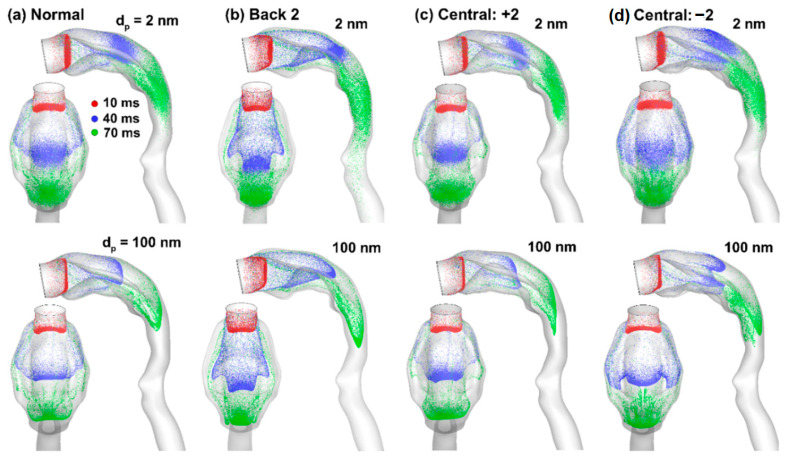
Aerosol transport across models with different tongue positions at 30 L/min: (**a**) normal tongue, (**b**) Back 2 (back-raised), (**c**) Central: +2 (central-tucked), and (**d**) Central: −2 (central-raised). Two particle sizes, 2 nm (upper panel) and 100 nm (lower panel) were considered.

Figure 5 shows the snapshots of aerosol swarms at three instants (10, 40, and 70 ms) after inhalation. Overall, similar patterns are observed between 2-nm and 100-nm aerosols, except that the 2-nm aerosols appear more dispersed owing to their higher diffusivity. In addition, the dispersion pattern increases over time, leading to no clear aerosol profiles at 70 ms. By contrast, the 100-nm aerosols still retain a well-defined front at 70 ms, regardless of the tongue positions (Figure 5a–d). This is reasonable in light of the concurrent low inertia and low diffusivity of 100-nm particles, which are presumed to follow airflows faithfully. Comparing the four models, the morphologies of the aerosol swarms are sensitive to the tongue postures. At 40 ms after inhalation (blue), the particles reached the middle oral cavity and exhibited distinct profiles due to tongue disruption, as evident in both the lateral and top views.

Complex wall-particle interactions occur as particles pass the protruding tongue. In Figure 5d, the central-tucked tongue elicited a triad-peak aerosol profile (from the top view) and a projecting peak near the mouth roof (lateral view) at 40 ms (blue). In the other three cases, the triad-peak profile is less apparent or not noticeable, indicating weak or delayed secondary flows towards the teeth-cheek lumen (Figure 5a–c, blue).

Differences in particle profiles are also noted among the models, even at 10 ms (red) before reaching the tongue, reflecting the global, holistic impact of local structural variations in internal flow problems. In contrast to the fast-moving aerosol front conveyed by the bulk flow, slow-moving particles are also noted near the airway wall. Even at 70 ms, there are still particles lingering near the mouth inlet (green, Figure 5a–d).

#### 3.1.3. Nanoparticle Deposition

**Figure 6:**
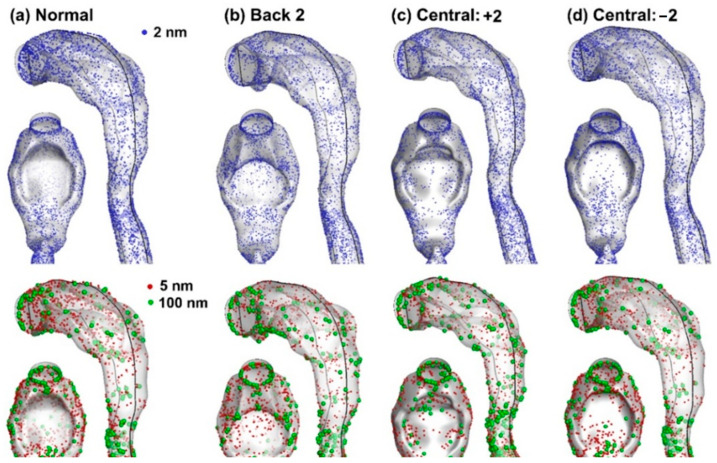
Surface deposition of nanoparticles due to different tongue positions: (**a**) Normal, (**b**) Back 2, (**c**) Central: +2, and (**d**) Central: −2. Different deposition patterns exist between different particle sizes: 2 nm (upper panel) vs. 5 nm (red, lower panel) vs. 100 nm (green, lower panel).

Surface deposition of inhaled nanoparticles in the four models is compared in Figure 6 for 2 nm, 5 nm, and 100 nm particles. Due to a decreasing diffusivity, the deposition distribution appears homogeneous for 2-nm particles and becomes increasingly more heterogeneous for 5-nm and 100-nm particles, as evidenced by the second row vs. the first row in Figure 6. Among the four models, elevated deposition in the posterior tongue was found in the case with the central-tucked tongue (Central: −2), which was consistent with the extensive recirculation zone, as observed in Figure 3d. Following this logic, we also observed elevated, but at a lesser level, deposition in the posterior tongue in the case with a neutral tongue, which likewise had an extensive recirculation behind it (Figure 3a). Note that all deposition distributions appear complex and lack distinct patterns (Figure 6); thus, it is challenging to establish the tongue posture-deposition relationships merely from an examination of the deposition distributions.

### 3.2. Training Dataset Preprocessing

#### 3.2.1. Deposition Data Evaluation

**Figure 7:**
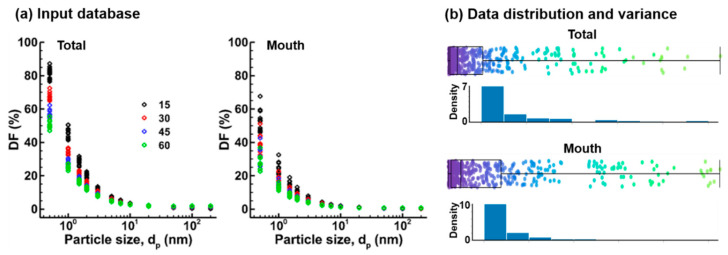
Training database of deposition fractions (DFs) from physiology-based simulations: (**a**) DF vs. particle size in the total oropharyngeal airway and mouth for different breathing conditions and tongue/glottis shapes; (**b**) highly skewed distributions of the DF data based on the arithmetic scale.

Figure 7a shows the semilogarithmic plots of the total and regional (mouth) deposition fractions (DFs) of 290 test cases. The data points are colored differently to represent different breathing rates (15, 30, 45, and 60 L/min), with each rate depicted by a unique color. In both plots, small particles exhibit a very high DF, which decreases sharply as particle size increases. Large variances are observed at the lower end of the nanoparticle size range (0.5–5 nm). For a given particle size, the total DFs are clustered into four groups specific to the flow rates (15, 30, 45, and 60 L/min), as illustrated in the right panel of Figure 7a. No significant differences were observed in the semilog DF plots for nanoparticles ranging from 7 nm to 200 nm, owing to their low DF values (Figure 7a). The same data for total and mouth DFs were also plotted in Figure 7b as the boxplot and histogram, providing a visual summary of the deposition data distribution and variance across particle sizes. In the histograms, the height of each bar corresponds to the frequency of deposition events, or the likelihood of deposition, within each size range. The DFs exhibit highly skewed distributions towards the lower end in both the total and mouth regions, which could adversely impact the performance of the surrogate model to be trained, including bias, learning inefficiency, and reduced accuracy. Thus, addressing the data skewness is needed to mitigate its effects on model training.

#### 3.2.2. Deposition Data in Log-Log Plot

**Figure 8:**
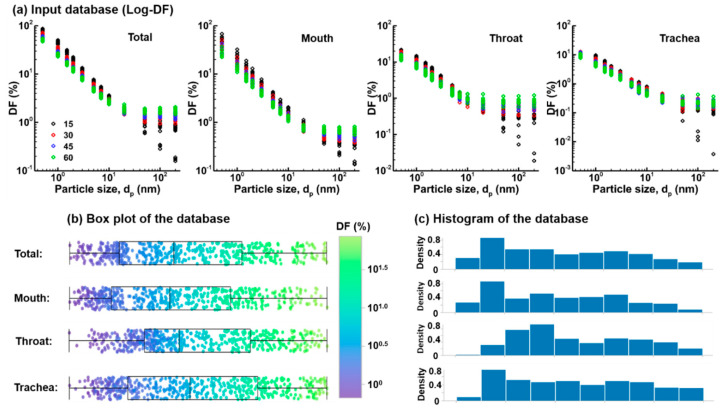
Preprocessing of the training database using the logarithmic scale: (**a**) DF vs. particle size in the total oropharyngeal airway, mouth, throat, and trachea for different breathing conditions and tongue/glottis shapes; (**b**) boxplot of the input data with approximately normal distributions based on the logarithmic scale, and (**c**) histogram of the database on the logarithmic scale.

Figure 8 displays the scatter plots of 290 DF values in the log-log scale, considering both the total and three subregions (mouth, throat, and trachea). The use of a log-log scale effectively spreads out the DF values clustered at the lower end, leading to a more balanced distribution across all particle sizes considered. Such skewness mitigation allows for a more linear representation of multivariate relationships between DFs and delivery factors like particle size, inhalation rate, etc. The log-log plots also help to visualize the U-shaped deposition behavior typically seen in aerosol science, where very small and large particles have higher DFs than mid-range sizes. As seen in Figure 8b, a bifurcation in DF values becomes noticeable at 50 nm and constantly increases at 100 nm and 200 nm. The deviating DFs all result from the lowest flow rate (10 L/min), indicating negligible inertia deposition mechanism at this flow rate. Particularly, in contrast to the lowest DF values for large nanoparticles at 10 L/min, the DFs at this flow rate are the highest for small particles among all flow rates considered, reflecting the dominant diffusion deposition at the lower end.

As a particle increases in size, its diffusivity decreases while its inertia increases. As a result, a cross point exists where the contributions from diffusion and inertia deposition mechanisms become equivalent. Moreover, a higher flow rate leads to a lower DF for small nanoparticles by reducing their residence time, but it leads to a higher DF for large nanoparticles due to increasing inertia (i.e., proportional to *v*^2^). Thus, the interactions between flow rate and particle size and their effect (total effect) on deposition should be considered in addition to their individual effects (i.e., main effects).

### 3.3. Surrogate Model Development and Validation

#### 3.3.1. Model Development

**Figure 9:**
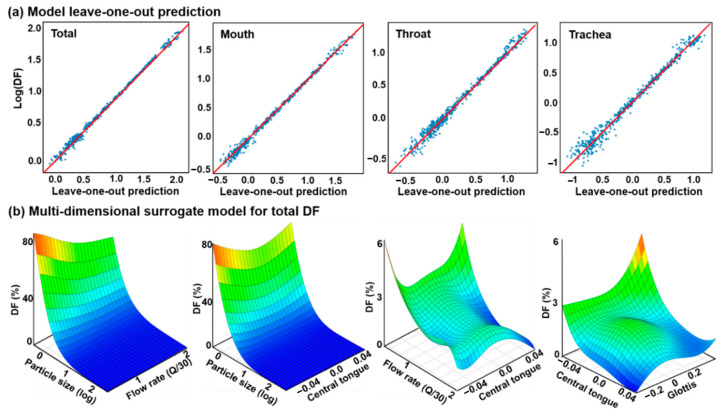
The surrogate predictive model (emulator) from the training database. (**a**) Leave-one-out cross-validation by comparing emulator predictions (red line) to the training data (blue dots). (**b**) 3D visualization of emulator predictions vs. input pair: (particle size and flow rate), (particle size and central tongue), (flow rate and central tongue), and (central tongue and glottis).

A surrogate predictive model, or emulator, was trained based on the dataset using Gaussian process regression and polynomial kernels in SmartUQ (Madison, WI, USA). Figure 9a shows the training accuracy of the newly developed emulators utilizing the technique of leave-one-out cross-validation. A close match was achieved between the emulator prediction (red line) and the training database (blue dots) for nanoparticle deposition in both the total and regional (mouth, throat, and trachea) airways.

It is noted that the surrogate model included the effects of five input factors. To visualize the model, 3D response surfaces were plotted vs. four pairs of input variables on a 50 × 50 grid, which exhibit very different response patterns (Figure 9b). The first panel in Figure 9b displays the response surface of the total DF as a function of particle size (d_p_) and flow rate (Q), which accurately captures the trend of a decreasing DF with increasing particle size and flow rate. A more dramatic decrease in DF with particle size than with flow rate was also captured. In contrast, the relationship between DF and the central tongue appears nonlinear, as shown in the second panel of Figure 9b, indicating a complex interplay between airflow, particle motion, and airway morphology. This complexity is even more pronounced when combined with the influences from the breathing conditions (the third panel) or other morphological factors (the fourth panel). The predictive surrogate model, or the trained emulator, provides an implicit tool that can accurately approximate the input-output relationship of the system and be used for future design exploration and analysis.

#### 3.3.2. Model Validation

**Figure 10:**
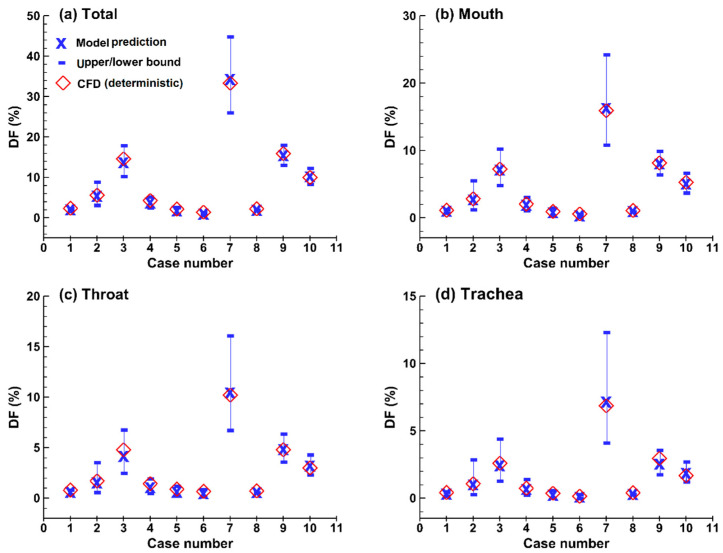
High predictive accuracy of the surrogate model demonstrated by comparison to deterministic (computational fluid dynamics, or CFD-predicted) results for ten randomly generated test cases (Table 2) that have not been included in the training database: (**a**) total DF, (**b**) mouth DF, (**c**) throat DF, and (**d**) trachea DF.

It is crucial to validate the surrogate model before applying it for future analysis. To evaluate whether the surrogate model was overfitted or not, ten test cases were randomly selected from the design space, as listed in Table 2, with their deposition fractions (DFs) being predicted using CFD and the surrogate model. Note that none of the ten cases have been seen by the training dataset. Figure 10 shows the CFD simulations (deterministic, red diamond symbols) in comparison to emulator predictions (probabilistic) with the mean (blue cross) and upper/lower bounds (blue hyphens). Good agreement was obtained between the emulator and CFD-predicted DFs for all the ten cases considered in both the total oropharyngeal airway (Figure 10a) and its three compartments (i.e., mouth, throat, and trachea), as illustrated in Figure 10b, Figure 10c, Figure 10d, respectively. Considering that the test cases in the training dataset had regularly distributed inputs, as shown in Figure 2a, the close CFD-emulator match for all randomized test cases in Figure 10 indicated that model overfitting was not a concern herein.

### 3.4. Sensitivity Analysis (SA) and Uncertainty Quantification (UQ)

#### 3.4.1. Input Sensitivity Analysis

**Figure 11:**
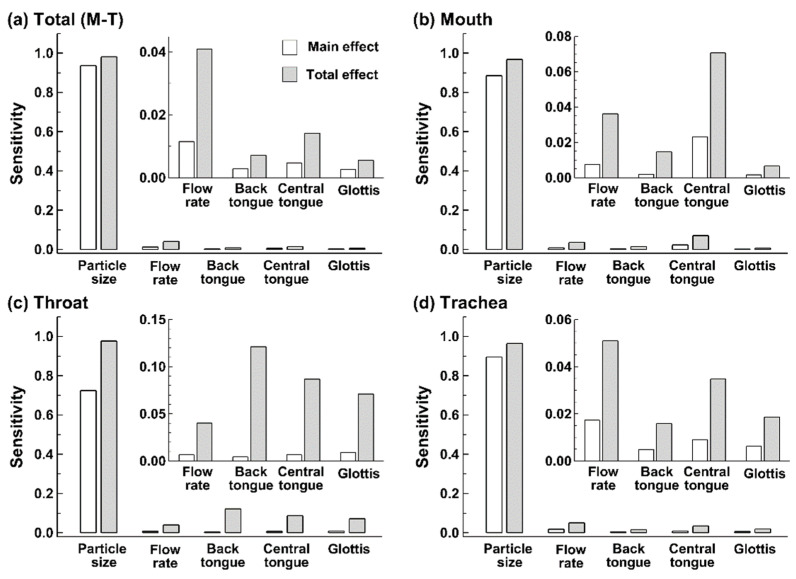
Sensitivity analysis (SA) of input variables in terms of aerosol (particle size), breathing (flow rate), and airway anatomy (back tongue, central tongue, and glottis) for inhalation dosimetry of inhaled nanoparticles in the (**a**) total mouth-throat (MT) airway, (**b**) mouth, (**c**) throat, and (**d**) trachea. Two ranges in the y-axis were used to highlight the relative importance among inputs.

Figure 11 and Table 3 display the sensitivity analyses of various input variables on nanoparticle deposition in different regions of the oropharyngeal airway: total (all regions), mouth, throat, and trachea. Recall that the main effect is the isolated effect of one factor on deposition, not taking into account any interactions with other factors, while the total effect considers all pathways through which one factor affects deposition and thus can be helpful in identifying causal relationships or deposition mechanisms. In all regions, particle size shows the highest main and total effects, indicating that it is the most influential factor in nanoparticle deposition. Moreover, the total effect is slightly larger than the main effect, suggesting relatively minor contributions from inputs other than the size-associated diffusion.

A comparison of factors other than the particle size is shown in the inset in each panel of Figure 11 and Table 3. The total effects are much larger than the main effects for all four factors (flow rate, back tongue, central tongue, and glottal area), indicating intensive interactions of the factor with others in regulating particle behaviors and fates. For instance, the flow rate affects not only the flow-particle residence time but also the particle inertia, flow regime in the core flow, and fluid-structure interactions in the near-wall region (i.e., flow boundary thickness, vortex shedding, wall shear, etc.). It is also noted that deposition in different regions has different sensitivity to the inputs. In this study, we observed that total deposition is most sensitive to the flow rate (Figure 11a), mouth deposition to the central tongue (Figure 11b), throat deposition to the back tongue (Figure 11c), and trachea deposition to the flow rate (Figure 11d).

#### 3.4.2. Deposition Uncertainty Quantification

**Figure 12:**
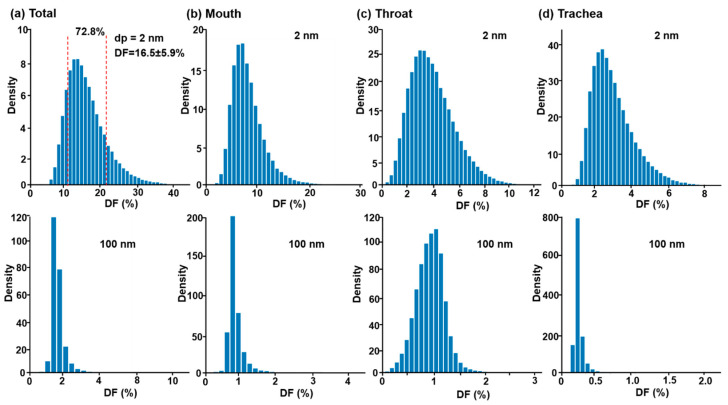
Uncertainty quantification (UQ) in DF due to the variability of five input factors (particle size, flow rate, central tongue, back tongue, and glottis, as listed in Table 4) in the (**a**) total oropharyngeal airway, (**b**) mouth, (**c**) throat, and (**d**) trachea. The upper panel shows the UQ for 2 nm aerosols, while the lower panel is for 100 nm aerosols.

Two examples of output uncertainty quantification (UQ) are presented in Figure 12 based on the trained surrogated model (emulator), i.e., for nanoparticles of 2 nm and 100 nm. The variances of the five inputs are listed in Table 4. For each particle size, 100,000 emulator-based Monta-Carlo simulations were performed to quantify the deposition uncertainty in the oropharyngeal airway and three subregions (mouth, throat, and trachea).

In Figure 12, the *x*-axis of the histogram represents the expected deposition rate, and the *y*-axis represents the probability of observing the expected deposition. For 2-nm particles (upper rows), all histogram distributions are approximately bell-shaped, suggesting the deposition remains largely normally distributed. Differences also exist among the four histograms in their symmetry and width. The right-skewness suggests an increased likelihood for higher dosages in the oropharyngeal airway and three subregions in practice when facing input uncertainties. The wide spread of the histogram indicates the possible occurrence of large deviations from the expected dose, for instance, 16.5% for the total deposition of 2-nm particles. In this case, the dose uncertainty is ±5.6% around the mean with a confidence level of 72.8% (Table 4 and Figure 12a). Similarly, with the same input uncertainties, the mean and uncertainty in sub-regional doses (mouth, throat, and trachea) were predicted as 8.2 ± 2.9%, 5.0 ± 1.5%, and 3.1 ± 1.2%, respectively (Table 4).

Distinct differences in histogram distributions are observed between 100-nm and 2-nm aerosols. The histogram widths for 100-nm particles are much narrower, with a steep drop on both sides. This is partially due to the low deposition rates for particles around 100 nm in size and represents low uncertainties in total and subregional deposition. On the other hand, the larger probability density values for 100-nm particles indicate more frequent observations of the expected doses.

## 4. Discussion

Variability in pulmonary drug delivery can stem from many factors, such as the airway geometry, the patient’s breathing pattern, device use, and aerosol properties. This study focused on the airway morphological effect on dosimetry of inhaled nanomedicine. A framework was developed that involved model reconstruction, CFD simulations, database preprocessing, surrogate model training, model validation against new simulations, and subsequently, sensitivity analysis and uncertainty quantification. In contrast to previous deterministic predictions with the output as one specific value, novel information on the input-output relation can be extracted from probabilistic analyses, as discussed below.

### 4.1. Input Sensitivity Analysis and Interaction-Associated Complexity

Quantifying inhalation dosimetry and associated uncertainty is a high-dimensional problem involving both quantitative variables (e.g., particle size, velocity, dimension) and unquantifiable factors (e.g., morphology). Not only do individual variables but also their interactions influence the final deposition and uncertainties. As a result, 290 test cases that covered the entire multivariate design space with all possible pairings were simulated, as demonstrated in Figure 2a. Both the main effect and total effect of each factor were quantified based on the design space hereof (Figure 11). It was no surprise that the nanoparticle size, which ranged from 0.5 to 200 nm with a diffusivity spanning five orders of magnitude (i.e., 2.17 × 10^−1^–2.29 × 10^−6^ cm^2^/s), contributed the highest variability to the deposition variability in both the entire oropharyngeal airway and three regions (mouth, throat, and trachea). The increment ratio of the total effect over the main effect was 2–3 orders of magnitude lower for particle size (0.05–0.35) than those for the flow rate (1.93–5.08) and morphological factors (1.13–25.87), as shown in Figure 11 and Table 3.

The high total-over-main increment ratio for the flow rate reflects the strong interactions with other factors, with the highest value in the throat (5.08) and the lowest in the trachea (1.93, Table 3). This observation is consistent with intensified inertia deposition in the throat vs. recirculation-enhanced mixing in the trachea, which reduced the deposition variation.

Considering the effect of tongue position, high sensitivity values are observed in the three regions of the mouth-throat geometry. Except for the particle size, the central tongue postures contribute the highest variability to the deposition in the mouth (Figure 11b), while the back tongue postures contribute the most to throat deposition (Figure 11c). The interaction between tongue postures and other factors is also significant. For the back tongue posture, the total-over-main increment ratio ranges from 1.46 (MT) to 25.87 (throat); for the central tongue posture, the ratio ranges from 1.96 (MT) to 11.95 (MT), as shown in Table 3.

Considering different regions, throat dosimetry was most sensitive to input variability among the three regions considered, as demonstrated by a higher *y*-range (0–0.15, insets, Figure 11c vs. Figure 11b,d). The total-over-main increment ratios in the throat (5.07–25.87) are also notably higher than in the mouth (2.04–6.15) and trachea (1.93–2.85), as listed in Table 3.

Sensitivity analysis helps identify the key input parameters that affect drug delivery efficiency, allowing researchers to optimize formulations, device designs, or delivery methods to enhance drug deposition in the targeted areas of the respiratory system. On the other hand, inputs with low sensitivity can be ruled out, which also indicates a wider design window with a similar confidence level in the output. By comparing the main and total effects, the interaction analysis can shed light on the complex mechanisms of drug deposition in the lungs. This knowledge can contribute to the development of more effective drug delivery strategies and technologies.

### 4.2. Output Uncertainty Quantification (UQ)

The ability to quantify drug delivery uncertainty is highly desirable in the evaluation of the therapeutic outcomes of medicines. The framework proposed in this study, as depicted in Figure 2d,e, has been demonstrated to be a good candidate for this aim. Two examples, using nanomedicines of 2 nm and 100 nm, were presented to illustrate the utility of surrogate modeling in deposition uncertainty quantification. For a given set of multivariate input variability, both the mean and standard deviation were obtained based on 100,000 samples using the trained surrogate model, as shown in Table 4. Moreover, the probability density functions vs. the deposition fraction (DF) were also quantified and visualized in Figure 12, clearly showing that the uncertainty in dosimetry for 2-nm aerosols was much higher than that for 100-nm aerosols.

Uncertainty quantification in the deposition of orally inhaled drug particles has great implications for improving the reliability, safety, and effectiveness of therapeutic interventions. By quantifying the uncertainties in delivered doses, developers can better understand the variability in patient response, leading to safer and more effective dosage recommendations. This reduces the risk of underdosing or overdosing, ensuring that patients receive the most beneficial amount of medication. Moreover, UQ enables the tailoring of drug delivery to individual patients by considering the variability in anatomy, physiology, and breathing patterns. This personalization ensures that each patient receives the most effective treatment based on their unique characteristics. From a regulatory perspective, demonstrating a thorough understanding of the uncertainties in drug delivery processes can facilitate regulatory approval. Regulatory bodies demand rigorous evidence of safety and efficacy, which UQ can provide by systematically addressing and quantifying uncertainties.

### 4.3. Limitations and Future Studies

This study can be further improved by considering additional influencing factors. First, only one original mouth-throat model was used to generate variants of the tongue and glottis. A large cohort of patient-specific, mouth-throat models can further verify or amend our understanding of the geometrical effect of orally inhaled medication dosimetry as well as the confidence level regarding the intersubject variability in device or medication bioequivalence (BE) [42,43,44,45,46]. Second, tidal breathing specific to the device and patient should be used to more accurately simulate the drug disposition distributions, even though steady flows have been shown to generate deposition distributions similar to those under corresponding tidal breathing conditions [47,48,49]. Third, all tongue variants were assumed to be static in this study, while they could be dynamic during the drug delivery, necessitating future studies to consider the dynamic motions of the tongue under tidal breathing [50,51]. Note that the morphology of a particle, like a sphere, rod, flexible fiber, disk, etc., can exert a notable effect on its transport and deposition, which should be considered in future studies [52,53,54,55,56].

For the mouth-throat model considered in this study, only morphological variations in the tongue posture and glottal aperture were considered. Important morphological factors include, but are not limited to, the oral cavity volume, mouth-throat curvature, pharynx constriction, and trachea diameter [57,58,59]. Moreover, the tracheobronchial (TB) region, middle lung, and acini (or alveolar region) should also be considered in future dosimetry uncertainty quantitation studies [60,61]. Structural variability or uncertainty in these regions not only affect the medication distribution; they themselves are the target of drug delivery. Thus, the predicted dose uncertainty in these regions will determine the confidence level in therapeutic outcomes or the bioequivalence compared to a counterpart device or medication [62]. Note that in recent years, machine learning has been actively explored in inhalation drug delivery that aimed to increase the prediction speed, identify causal relationships, and quantify dosimetry uncertainty [63,64,65,66,67].

## 5. Conclusions

In summary, a surrogate model for upper airway deposition of orally inhaled nanoparticles was trained based on a CFD database using Gaussian process regression and polynomial kernels. Among the five inputs considered, the particle size was observed to be the most sensitive factor on nanoparticle deposition, while the flow rate and tongue posture exert similar levels of impact. Particularly, the central tongue mainly affected mouth deposition, the back tongue affected throat deposition, while the flow rate affected the trachea and total deposition. High levels of interaction effects were observed among input parameters, which were often neglected in deterministic simulations. The utility of the surrogate model for nanomedicine dosimetry uncertainty quantification was demonstrated for 2-nm and 100-nm aerosols, providing the mean, standard deviation, and probability density distribution. CFD-based surrogate modeling can provide more realistic dosimetry estimation in the face of input variability or uncertainty, thus allowing for probability-informed decision-making.

## Figures and Tables

**Table 1:** Morphological dimensions of various mouth-throat-tongue models. C: Central tongue.

	Mean	Back 1	Back 2	C−2	C−1	C+1	C+2
Volume, V (cm^3^)	49.8	48.3	47.2	44.6	47.1	51.7	53.9
Vol change (%)	0.0	−2.96	−5.20	−10.48	−5.54	3.79	8.17
Surface area, A (cm^2^)	107.8	106.4	108.0	112.0	109.2	107.3	106.1
Surface area change (%)	0.0	−1.28	0.23	3.92	1.26	−0.48	−1.55

**Table 2:** Test cases with randomly generated operating conditions.

Variables	1	2	3	4	5	6	7	8	9	10
Flow rate (L/min)	46.7	20.6	32.9	48.5	56.4	41.5	17.4	45.1	43.4	58.5
Particle size (nm)	10.3	5.7	2.0	5.7	13.5	70.2	1.3	11.0	1.7	2.3

**Table 3:** The main and total effects of the inputs on deposition in different regions.

		Particle Size	Flow Rate	Back Tongue	Central Tongue	Glottis Area
All regions	Main	0.93706	0.01156	0.00288	0.00476	0.00260
	Total	0.98178	0.04095	0.00709	0.01411	0.00555
	Ratio *	0.05	2.54	1.47	1.96	1.13
Mouth	Main	0.88512	0.00758	0.00204	0.02321	0.00164
	Total	0.96728	0.03610	0.01458	0.07058	0.00661
	Ratio *	0.09	3.76	6.15	2.04	3.03
Throat	Main	0.72473	0.00661	0.00451	0.00670	0.00908
	Total	0.97685	0.04019	0.12131	0.08676	0.07096
	Ratio *	0.35	5.08	25.90	11.95	6.81
Trachea	Main	0.89502	0.0174	0.0049	0.00904	0.00624
	Total	0.96469	0.0510	0.0159	0.03486	0.01868
	Ratio *	0.08	1.93	2.24	2.86	1.99

* Ratio: the increment ratio of the total effect over the main effect.

**Table 4:** Input variability and output uncertainty with 100,000 samplings.

Inputs with Normal Distributions					Predicted Output (DF) Uncertainty (%)			
Flow (L/min)	Particle Size (nm)	Back_T ∆V/V_0_ (%)	Central_T ∆V/V_0_ (%)	Glottis ∆A/A_0_	Total	Mouth	Throat	Trachea
30 ± 9	2 ± 1.25	−1.5 ± 0.5	0 ± 1.0	0 ± 0.05	16.5 ± 5.6	8.2 ± 2.9	5.0 ± 1.5	3.1 ± 1.2
30 ± 9	100 ± 30	−1.5 ± 0.5	0 ± 1.0	0 ± 0.05	1.5 ± 0.5	0.6 ± 0.4	0.9 ± 0.3	0.2 ± 0.1

## Data Availability

Data are contained within the article.
